# Effects of Geographical and Climatic Factors on the Intrinsic Water Use Efficiency of Tropical Plants: Evidence from Leaf ^13^C

**DOI:** 10.3390/plants12040951

**Published:** 2023-02-20

**Authors:** Xiaoyan Lin, Bingsun Wu, Jingjing Wang, Guoan Wang, Zixun Chen, Yongyi Liang, Jiexi Liu, Hao Wang

**Affiliations:** 1 School of Forestry, Hainan University, Haikou 570228, China; 2 Rubber Research Institute, Chinese Academy of Tropical Agricultural Sciences, Haikou 571101, China; 3 Opening Project Fund of Key Laboratory of Biology and Genetic Resources of Rubber Tree/State Key Laboratory Breeding Base of Cultivation and Physiology for Tropical Crops/Danzhou Investigation and Experiment Station of Tropical Crops, Ministry of Agriculture and Rural Affairs, Danzhou 571700, China; 4 Beijing Key Laboratory of Farmland Soil Pollution Prevention and Remediation, Department of Environmental Sciences and Engineering, College of Resources and Environmental Sciences, China Agricultural University, Beijing 100193, China; 5 National Key Laboratory of Plant Molecular Genetics, CAS Center for Excellence in Molecular Plant Sciences, Institutes of Plant Physiology and Ecology, Shanghai 200032, China; 6 College of International Studies, Yangzhou University, Yangzhou 225009, China

**Keywords:** water use efficiency, carbon isotope composition, leaf carbon content, climate change

## Abstract

Understanding the water use efficiency (WUE) and adaptation strategies of plants in high-temperature and rainy areas is essential under global climate change. The leaf carbon content (LCC) and intrinsic WUE of 424 plant samples (from 312 plant species) on Hainan Island were measured to examine their relationship with geographical and climatic factors in herbs, trees, vines and ferns. The LCC ranged from 306.30 to 559.20 mg g^−1^, with an average of 418.85 mg g^−1^, and decreased with increasing mean annual temperature (MAT). The range of intrinsic WUE was 8.61 to 123.39 μmol mol^−1^ with an average value of 60.66 μmol mol^−1^. The intrinsic WUE decreased with increasing altitude and relative humidity (RH) and wind speed (WS), but increased with increasing latitude, MAT and rainy season temperature (RST), indicating that geographical and climatic factors affect the intrinsic WUE. Stepwise regression suggested that in tropical regions with high temperature and humidity, the change in plant intrinsic WUE was mainly driven by WS. In addition, the main factors affecting the intrinsic WUE of different plant functional types of plants are unique, implying that plants of different plant functional types have distinctive adaptive strategies to environmental change. The present study may provide an insight in water management in tropical rainforest.

## 1. Introduction

Plant water use efficiency (WUE) reflects the balance between carbon assimilation by photosynthesis and water lost by stomata [1]; it is an important indicator for studying plant water use strategies. The world is experiencing rapid climate change. The average temperature in the second half of this century increased by approximately 3 °C compared with the temperature in 1850–1900, and the frequency of extreme climate events caused by climate warming obviously increased [2]. The precipitation in high-latitude areas has increased, while the precipitation in tropical areas has decreased [3], and the global rainfall pattern has changed dramatically [4]. Moreover, the atmospheric CO_2_ concentration (c_a_) increased from 277 ppm in 1750 to 413 ppm in 2020, and the annual average rate of increase of atmospheric CO_2_ concentration in the past decade was 2.40 ppm [2]. Climate changes have already caused variations in the living conditions of vegetation [5]. As a result, plants have to form some physiological adaptation mechanisms in response to climate change. The change in WUE by adjusting plant stomatal conductance (g_s_) and CO_2_ assimilation rate (A) [5] is one of the adaptation mechanisms by which plants can better cope with climate change [6]. Therefore, investigating the variations in plant WUE under climate change will serve to deepen the understanding of the response of vegetation to global climate change.

There have been several indexes used to evaluate WUE, including the WUE at yield level (= yield/water consumption), the instantaneous WUE (= CO_2_ assimilation rate/transpiration rate, A/E) and the intrinsic WUE (= CO_2_ assimilation rate/stomatal conductance, A/g_s_). Plant carbon isotopes have been widely used to indicate the intrinsic WUE [7,8,9,10,11]. The carbon isotope discrimination (Δ^13^C) in plant leaves depends on the ratio of intercellular and environmental CO_2_ concentrations (c_i_/c_a_), and this ratio is regulated by g_s_ and A [4,12]. The intrinsic WUE of plants is defined by the ratio of A to g_s_, which is also associated with c_i_/c_a_ [6]. Therefore, foliar Δ^13^C is a useful proxy of plant intrinsic WUE, and there is a negative correlation between leaf Δ^13^C and intrinsic WUE [13,14,15]. In addition, leaf Δ^13^C represents the time-integrated plant intrinsic WUE during leaf formation [16], and thus it can better represent the long-term plant water use status.

Climatic factors, such as temperature, humidity and light intensity affect the intercellular CO_2_ concentration by affecting leaf stomatal conductance and photosynthetic carboxylase, thus influencing plant WUE [16,17,18]. Camarero et al. (2021) [6] and Driscoll et al. (2020) [19] found that the intrinsic WUE of plants was positively correlated with temperature. Kørup et al. (2017) [20] and Mathias and Thomas (2021) [21] illustrated that there was a significantly negative correlation between plant intrinsic WUE and rainfall. Olson et al. (2020) [22] and Guo et al. (2018) [23] proposed that relative humidity affected plant intrinsic WUE. Zhang et al. (2020) [24] found that wind speed affected the boundary layer of the air on the leaf surface, which decreased the resistance for gas exchange and the exchange of CO_2_ and H_2_O between the leaf interior and ambient atmosphere, thereby influencing the leaf WUE. In addition, since climatic factors change with geographical factors, intrinsic WUE changes with geographical factors [25,26]. Although many previous works have focused on the relationships between plant WUE and climatic factors [15], most of these studies were concentrated mainly on arid and semiarid climate regions, or the research objects were mostly one or several plants [19,22,27]; in contrast, few studies have focused on tropical regions with high temperature and rainfall. Previous studies have shown significant differences in intrinsic WUE between different species among habitats, indicating that intrinsic WUE and its responses to climate change in different species in different areas may be different [15]. There is a lack of integrated research on the WUE response of different plant functional types to climate change in tropical areas. However, the current warming rate in tropical areas is extraordinarily fast [28], and the impact of climate change on plants in tropical areas may be more serious than that in other areas [1]. Therefore, it is of great significance to study the response of leaf WUE to climate change in tropical regions.

In this study, plant samples were collected from Hainan Island, China, and the leaf carbon content (LCC) and leaf carbon isotope ratio (δ^13^C) were measured as indicators of leaf intrinsic WUE to investigate the relationship between the intrinsic WUE of different functional types and geographical and climatic factors in tropical regions. Our objectives were to explore the response mechanism of LCC and leaf intrinsic WUE to climate change and to improve the understanding of plant adaptation strategies in tropical regions.

## 2. Results

### 2.1. Characteristics of LCC and Intrinsic WUE

**Figure 1:**
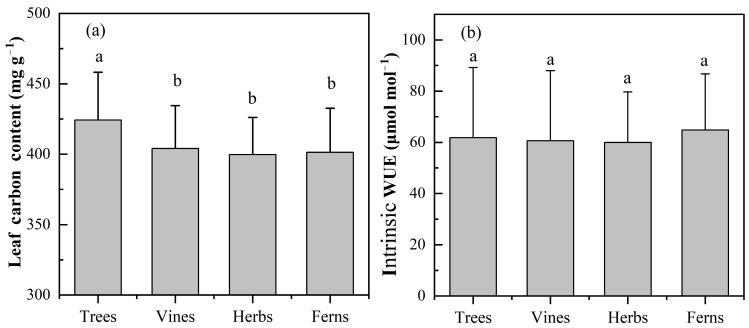
The mean leaf carbon content (LCC) and the intrinsic water use efficiency (intrinsic WUE) of trees, vines, herbs and ferns. (**a**) Leaf carbon content; (**b**) Intrinsic water use efficiency. Different letters indicate significant differences between different plant functional types (*p* < 0.05). Boxes and error bars represent the mean values and standard errors.

The LCC ranged from 306.30 to 559.20 mg g^−1^ with a mean value of 418.85 mg g^−1^. One-way analysis of variance (ANOVA) showed that there were significant differences in LCC across plant functional types (Figure 1a, *p* < 0.05). In detail, the LCC of trees (424.21 ± 38.32 mg g^−1^) was significantly higher than that of other plant functional types (*p* < 0.01 for vines and herbs, *p* < 0.05 for ferns, Figure 1a). However, there were no significant differences in LCC among vines (404.04 ± 30.89 mg g^−1^), herbs (398.87 ± 26.13 mg g^−1^) and ferns (401.34 ± 3.30 mg g^−1^) in the study area (Figure 1a). Multi-factor ANOVA analyses suggested that plant functional types and the interaction of location and altitudinal range played a significant role in LCC (*p* < 0.05, Table 1).

The intrinsic WUE ranged from 8.61 to 123.39 μmol mol^−1^ with an average value of 60.66 μmol mol^−1^. There were no significant differences in the intrinsic WUE of the different plant functional types (Figure 1b). Multi-factor ANOVA analyses indicated that plant functional types, location and altitudinal range had significant impacts on intrinsic WUE (*p* < 0.05, Table 1); however, the effect of the interaction of these three factors on intrinsic WUE was insignificant. 

### 2.2. Relationships among LCC, δ^13^C and Geographical and Climatic Factors

**Figure 2:**
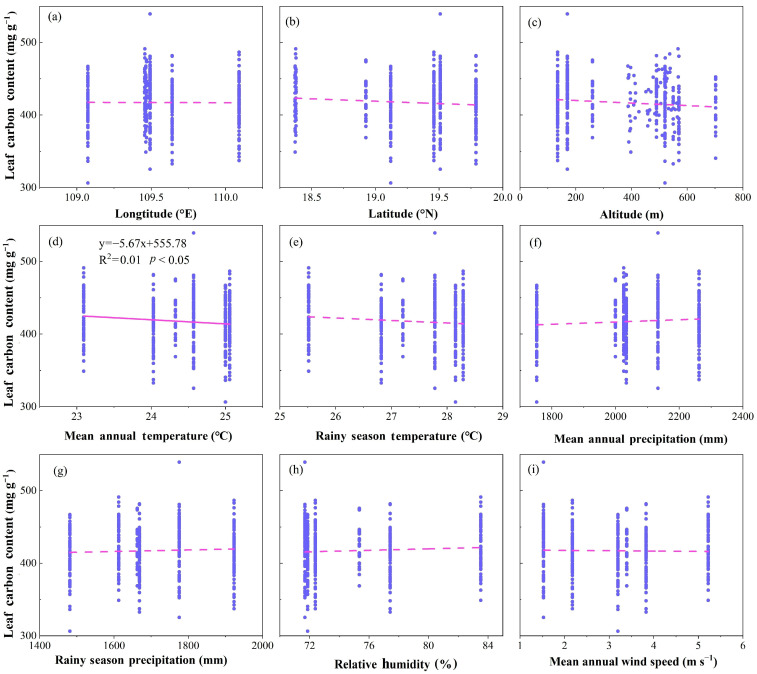
Changes in leaf carbon content (LCC, mg g^−1^) with geographical and climatic factors in all samples. (**a**) LCC vs. longitude; (**b**) LCC vs. latitude; (**c**) LCC vs. altitude; (**d**) LCC vs. mean annual temperature; (**e**) LCC vs. rainy season temperature; (**f**) LCC vs. mean annual precipitation; (**g**) LCC vs. rainy season precipitation; (**h**) LCC vs. relative humidity; (**i**) LCC vs. mean annual wind speed. The scatter points in the figure represent the measured C content of plant leaves. The dotted line indicates no significant correlation between LCC content and geographical and climatic factors (*p* > 0.05). The solid line indicates that the correlation between LCC content and climatic factors is significant (*p* < 0.05).

Linear regression analysis showed that LCC was significantly negatively correlated with mean annual temperature (MAT, R^2^ =0.01, *p* < 0.05, Figure 2f), but had no significant relationship with other geographical and climatic factors (*p* > 0.05, Figure 2). 

**Figure 3:**
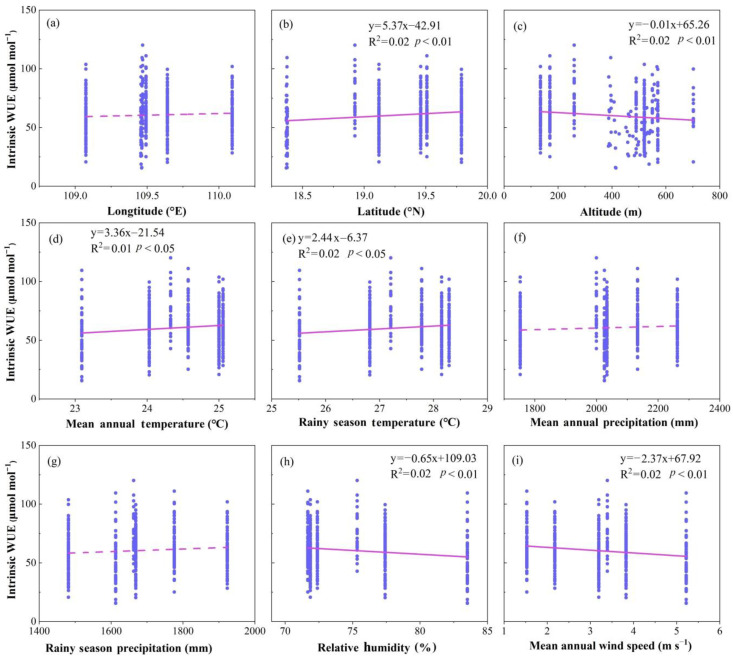
Changes in intrinsic water use efficiency (intrinsic WUE) with geographical climatic factors in all samples. (**a**) Intrinsic WUE vs. longitude; (**b**) intrinsic WUE vs. latitude; (**c**) intrinsic WUE vs. altitude; (**d**) intrinsic WUE vs. mean annual temperature; (**e**) intrinsic WUE vs. rainy season temperature; (**f**) intrinsic WUE vs. mean annual precipitation; (**g**) intrinsic WUE vs. rainy season precipitation; (**h**) intrinsic WUE vs. relative humidity; (**i**) intrinsic WUE vs. mean annual wind speed. The scatter points in the figure represent the measured intrinsic WUE of plant leaves. The dotted line indicates that the correlation between intrinsic WUE and climatic factors is not significant (*p* > 0.05). The solid line indicates that the correlation between leaf intrinsic WUE and climatic factors is significant (*p* < 0.05).

The leaf intrinsic WUE was significantly positively correlated with latitude (R^2^ = 0.02, *p* < 0.01, Figure 3b), MAT (R^2^ = 0.02, *p* < 0.05, Figure 3d) and rainy season temperature (RST, R^2^ = 0.02, *p* < 0.05, Figure 3b,d,e), and it was significantly negatively related to altitude (R^2^ = 0.02, *p* < 0.01, Figure 3c), relative humidity (RH, R^2^ = 0.02, *p* < 0.01, Figure 3h) and wind speed (WS, R^2^ = 0.02, *p* < 0.01, Figure 3i). In addition, there was no significant correlation between intrinsic WUE and longitude, mean annual precipitation (MAP) and rainy season precipitation (RSP, all *p* > 0.05, Figure 3). Multiple linear regression analysis showed that only 8.3% of the variability in the intrinsic WUE could be explained as a linear combination of these nine factors (R^2^ = 0.083, *p* < 0.01, Table 2). Stepwise regression analysis revealed that only WS drove the change in intrinsic WUE (R^2^ = 0.024, *p* < 0.01, Table 2).

### 2.3. Relationships among LCC, Intrinsic WUE and Geographical and Climatic Factors of Different Plant Functional Types

**Figure 4:**
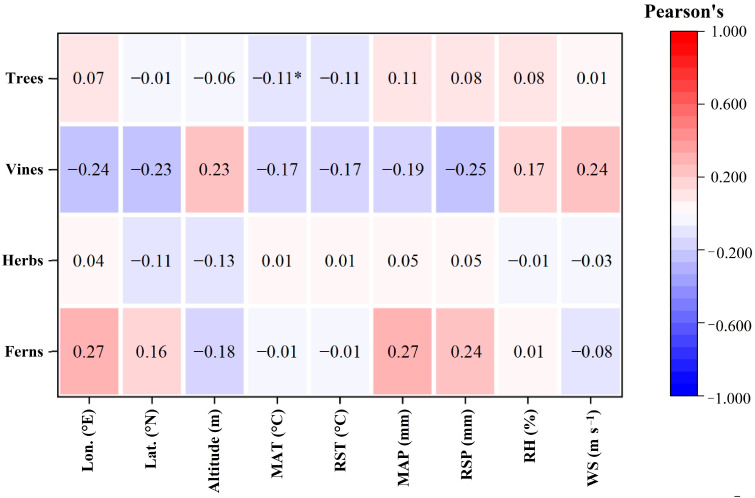
Pearson correlation coefficient of LCC in different plant functional types. * indicates significant correlations (*p* < 0.05). The color in the figure represents the correlation coefficient, with red indicating a positive correlation and blue indicating a negative correlation. Lon., longitude; Lat., latitude; MAT, mean annual temperature; RST, rainy season temperature; MAP, mean annual precipitation; RSP, rainy season precipitation; RH, relative humidity; WS, wind speed.

There was no significant correlation between the LCC in herbs, vines and ferns and the nine geographical climatic factors (*p* > 0.05, Figure 4). For trees, LCC was only negatively correlated only with MAT (*p* < 0.05, Figure 4).

**Figure 5:**
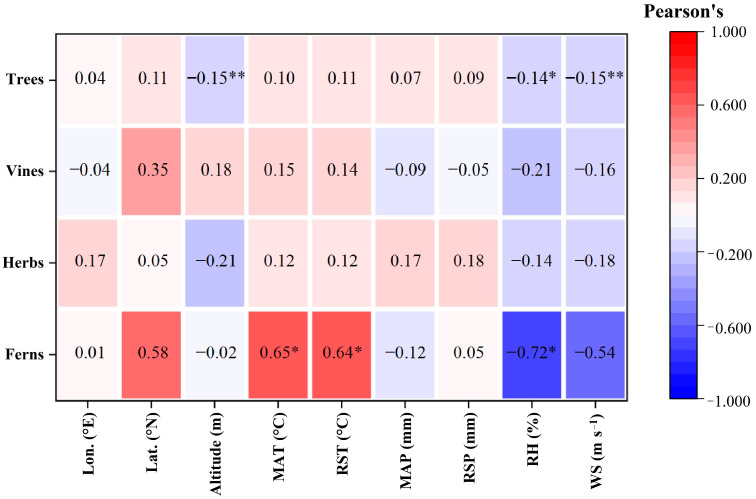
Pearson correlation coefficient of leaf intrinsic WUE in different plant functional types. * and ** indicate significant correlations at *p* < 0.05 and *p* < 0.01, respectively. The color in the figure represents the correlation coefficient, with red indicating a positive correlation and blue indicating a negative correlation. Lon., longitude; Lat., latitude; MAT, mean annual temperature; RST, rainy season temperature; MAP, mean annual precipitation; RSP, rainy season precipitation; RH, relative humidity; WS, wind speed.

The intrinsic WUE of trees was significantly negatively correlated with altitude, RH and WS (*p* < 0.05 for RH, *p* < 0.01 for altitude and WS, Figure 5) but had no significant relationship with the other factors (*p* > 0.05, Figure 5). There was no significant correlation between the intrinsic WUE and the nine geographical and climatic factors in herbs and vines (*p* > 0.05, Figure 5). Multiple linear regression analysis showed that only 8.1% of the variability in the intrinsic WUE could be explained as a linear combination of these nine factors in woody plants (R^2^ = 0.081, *p* < 0.01, Table 2). Stepwise regression analysis revealed that only altitude drove the change in intrinsic WUE in woody plants (R^2^ = 0.024, *p* < 0.01, Table 2). The intrinsic WUE of ferns was negatively correlated with RH (*p* < 0.05, Figure 5), but was positively related to MAT and RST (*p* < 0.05, Figure 5). Multiple linear regression analysis showed that 81% of the variability in the intrinsic WUE could be explained as a linear combination of these nine factors in ferns (R^2^ = 0.810, *p* < 0.05, Table 2). Stepwise regression analysis revealed that altitude and RH drove the change in intrinsic WUE in woody plants (R^2^ = 0.766, *p* < 0.01, Table 2).

## 3. Discussion

### 3.1. Characteristics of LCC and Its Relationship with Geographical and Climatic Factors in the Study Area

In the study area, the LCC ranged from 306.30 to 559.20 mg g^−1^ (Figure 1a) with a mean value of 418.85 mg g^−1^, which was lower than the LCC at the on a global scale [29], that in central and western China [30] and that in subtropical regions [31]. Moreover, the mean LCCs of trees and herbs were 424.2 mg g^−1^ and 399.7 mg g^−1^, respectively (Figure 1a), and these results were also lower than the results of a previous study [30,31,32]. The lower LCC may be caused by the higher temperatures on Hainan Island. Chapin et al. (2011) [33] found that the LCC of coniferous forests grown in cold environments was significantly higher than that of evergreen broad-leaved tree species. To improve cold resistance, the content of nonstructural carbon in plants, such as starch, low molecular weight sugar and stored lipids, must increase to balance the osmotic pressure of cells under low temperature [34,35], which might result in high LCC. The annual mean temperature on Hainan Island is high, and there is no frost throughout the year. Moreover, the dominant tree species in the study area are mainly evergreen broad-leaved forests. Therefore, the mean LCC in the study area was lower than that in other areas.

The present study showed that the LCC on Hainan Island was significantly negatively correlated with MAT (*p* < 0.05, Figure 2d) but had no significant correlation with the other factors (Figure 2). As mentioned above, a low temperature leads to the increase of in LCC by stimulating the synthesis of nonstructural carbon in plants [34,35]. In addition, a high temperature may restrain the activity of photosynthetic enzymes [36], resulting in a decrease of photosynthetic rate [37], and thus a decrease in LCC. 

The LCC in the study area had no significant correlation with other geographical and climatic factors (*p* > 0.05, Figure 2), which was different from the results of many previous studies [32,33,38,39,40] that suggested that geographical and climatic factors including longitude, latitude, altitude, precipitation, relative humidity and wind speed had significant effects on LCC. However, these effects were found at the global scale and in other climatic zones. Our study sites were located in a tropical region with high temperature and precipitation. The climate in tropical regions may be more favorable for plant growth than that in other regions; thus, the LCC in plants may be less sensitive to climatic change. Therefore, no relationship was found between the LCC and geographical and climatic factors except for temperature. 

### 3.2. Characteristics of LCC and Its Relationship with Geographical and Climatic Factors in Different Functional Types of Plants

Significant differences in LCC were found among different plant functional types (*p* < 0.05, Figure 1a). The LCC of trees was significantly higher than that of vines, herbs and ferns, which was consistent with the research of He et al. (2006) [30]. In general, the photosynthetic capacity of trees is higher than that of shrubs and herbs because of the greater photosynthetic quantum intensity received by trees. Thus, trees accumulate more carbon. In addition, trees contain a large number of carbon-rich compounds, such as lignin, tannin and structural carbohydrates, which may lead to a higher LCC in trees [41,42].

The LCC of trees was only significantly negatively correlated only with the MAT (*p* < 0.05, Figure 4), and had no significant relationship with other factors. This result was consistent with the results for all samples. There was no significant correlation between the LCC and geographical and climatic factors in herbs, vines and ferns (*p* > 0.05, Figure 4). Because of their shallow root system, herbs are more affected by changes in soil nutrient conditions than are woody plants [43]. Due to the high carbon assimilation rate per unit mass, great light energy absorption and utilization capacity [44,45] and well-developed root systems [46], the photosynthetic capacity of vines increases significantly with increasing soil nutrients [44,47]. Therefore, the main factors affecting the LCC of herbs and vines may be soil nutrients, rather than geographical and climatic factors. During fern growth, the opening and closing leaf stomata are affected mainly by red light [48]; thus, the LCC in ferns may also be less sensitive to geographical and climatic factors.

### 3.3. Characteristics of Intrinsic WUE and Its Relationship with Geographical and Climatic Factors in the Study Area

The range of intrinsic WUE was from 8.61 to 123.39 μmol mol^−1^ with an average value of 60.66 μmol mol^−1^ (Figure 1b), which was lower than the result from global, arid and semiarid climate regions [49,50]. A previous study showed that the amount of water loss by plants often exceeds the amount of carbon accumulation by three orders of magnitude for terrestrial plants [14]. Thus, plant growth is mainly affected by water limitation. With increasing water supply, plants tend to open their stomata to absorb more CO_2_, which leads to a higher the ratio of intercellular and environmental CO_2_ concentrations (c_i_/c_a_). Previous studies have suggested that plant intrinsic WUE was negatively correlated with c_i_/c_a_ [6,16,51]. As a result, the intrinsic WUE of plants under better water conditions is significantly lower than that under arid environments [20,49,51]. Compared with the study sites at the global scale and in arid and semiarid regions, the abundant water conditions on Hainan Island are more suitable for plant growth, leading to a lower intrinsic WUE than that obtained for global, arid and semiarid plants.

Many environmental factors affecting leaf intrinsic WUE [16]. The leaf intrinsic WUE in Hainan Island was significantly positively correlated with MAT (R^2^ = 0.02, *p* < 0.05, Figure 3d) and RST (R^2^ = 0.02, *p* < 0.05, Figure 3e). Camarero et al. (2014) [6] also reported that the intrinsic WUE was positively related to temperature. Under a high-temperature environment, the increase in temperature leads to a rapid loss of plant water by transpiration. Therefore, plants will close their stomata to avoid excessive water loss, which will lead to a sharp decrease in the leaf CO_2_ exchange rate with increasing temperature [19,52]. Therefore, the c_i_/c_a_ decreases with temperature, resulting in an increase in intrinsic WUE. As mentioned above, the global change in temperature is within approximately 3 °C [2]. The temperature span in the present study was 6.51 °C, which was greater than the global temperature change. Thus, our results suggested that global change in temperature would have a significant impact on the intrinsic WUE on Hainan Island.

There was no significant correlation between the intrinsic WUE and MAP and between the intrinsic WUE and RSP (*p* > 0.05, Figure 3f,g), suggesting that precipitation has a weak effect on the intrinsic WUE on Hainan Island. This result was different from the negative correlation between MAP and intrinsic WUE found in other studies [20,49,51]. Precipitation affects the intrinsic WUE by adjusting stomatal conductance. However, a previous study suggested that precipitation will no longer affect stomatal conductance, and thus intrinsic WUE, when it exceeds approximately 1800 mm [53]. On Hainan Island, the precipitation amount is higher than this value in most regions. Therefore, the change in precipitation in the study area had little effect on intrinsic WUE. 

In the study area, the intrinsic WUE was significantly negatively correlated with RH (R^2^ = 0.02, *p* < 0.01, Figure 3h), which was also been found in some previous studies [22,50]. The increase in RH relieves the water stress in plants, which promotes an increase in stomatal conductance. Therefore, the c_i_/c_a_ increases, resulting in lower intrinsic WUE.

A negative correlation was found between the intrinsic WUE and WS (R^2^ = 0.02, *p* < 0.01, Figure 3i). Wind speed affects the gas exchange in plant leaves by regulating the boundary layer of the air on the leaf surface [24,54]. The increasing wind speed reduces the thickness of the boundary layer, leading to a lower diffusion resistance for H_2_O and CO_2_. Therefore, the c_i_/c_a_ increases and the intrinsic WUE decreases with increasing wind speed. 

**Figure A1:**
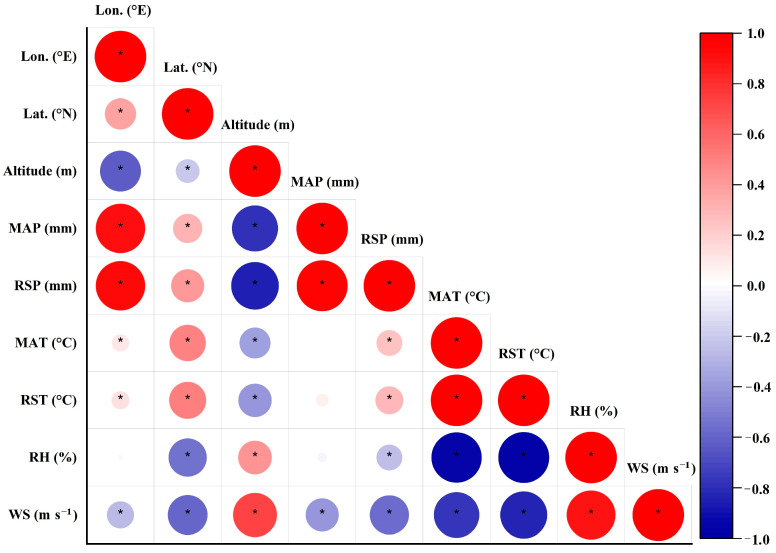
Pearson correlation coefficient of geographic and climatic factors. * indicates significant differences at *p* < 0.05. The circle in the figure represents the correlation coefficient, red represents positive correlation, blue represents negative correlation. Lon., longitude; Lat., latitude; MAT, mean annual temperature; RST, rainy season temperature; MAP, mean annual precipitation; RSP, rainy season precipitation; RH, relative humidity; WS, wind speed.

There was no significant correlation between the intrinsic WUE and longitude in the study area (*p* > 0.05, Figure 3a); however, the intrinsic WUE in the study area was positively correlated with latitude (R^2^ = 0.02, *p* < 0.01, Figure 3b) and negatively correlated with altitude (R^2^ = 0.02, *p* < 0.01, Figure 3c). In general, climatic factors change with geographical factors, resulting in the close relationship between intrinsic WUE and geographical factors. The lack of correlation between the intrinsic WUE and longitude may be caused by the relatively lower longitude span in the study area. The positive correlation between the intrinsic WUE and latitude may be caused by the negative links between latitude and WS and between latitude and RH (Figure A1). The negative relationship between altitude and intrinsic WUE can be explained by the negative correlation between temperature and intrinsic WUE (Figure A1). 

Collecting samples along the spatial gradient to study the relationship between intrinsic WUE and geographical and climatic factors will inevitably cause complex results due to the high collinearity of these factors. A high degree of collinearity between the geographical and climatic factors was also found on Hainan Island (Figure A1). Therefore, we conducted a stepwise regression analysis to determine the main factors influencing the intrinsic WUE. The results showed that only WS entered the model (Table 2), suggesting that WS was the key factor affecting plant intrinsic WUE on Hainan Island.

### 3.4. Characteristics of Leaf Intrinsic WUE and Its Relationship with Geographical and Climatic Factors in Different Plant Functional Types

There was no significant difference in the intrinsic WUE between different plant functional types in the study area (*p* > 0.05, Figure 1b). Peñuelas et al. (1999) [27] found that the intrinsic WUE of trees was significantly higher than that of herbs by measuring the leaf *δ*^13^C of four main plants growing in Spain. Woody plants have a longer water transport pathway due to higher plant height; thus, they have a higher intrinsic WUE than herbs. However, there was no significant difference in the intrinsic WUE between different plant functional types of plants on Hainan Island. This result may be associated with abundant water resources on Hainan Island. Because of high precipitation and air humidity, the surface soil water is sufficient to support for the growth of different functional types of plants, which may lead to no significant difference in the intrinsic WUE between different plant functional types.

There was a significantly negative correlation between the intrinsic WUE of trees and altitude, RH and WS (*p* < 0.01, Figure 5). Stepwise regression analysis showed that only altitude entered the model (Table 1). With increasing altitude, atmospheric pressure decreases, and the CO_2_ partial pressure decreases. Previous studies have illustrated that intrinsic WUE increases with CO_2_ partial pressure [19,21]. Therefore, the intrinsic WUE of trees decreased with increasing altitude. 

The intrinsic WUE in ferns was positively correlated with MAT and RST (*p* < 0.05, Figure 5), and negatively related to RH (*p* < 0.05, Figure 5). Stepwise regression analysis suggested that altitude and RH were the key factors affecting the intrinsic WUE in ferns (Table 2). In particular, the R^2^ in the regression model of the intrinsic WUE of ferns was much larger than that in the other models (Table 2), implying that the sensitivity of intrinsic WUE in ferns to geographical and climatic factors was much higher than that in other plant functional types.

There was no significant correlation between the intrinsic WUE and geographical and climatic factors in herbs and vines (*p* > 0.05, Figure 5). As mentioned above, herbs are more affected by changes in soil nutrient conditions than are woody plants because of the shallow root system [43]; similarly, the photosynthetic capacity of vines is primarily affected by soil nutrients [44,47]. Therefore, geographical and climatic factors may have little effect on the intrinsic WUE of herbs and ferns.

## 4. Materials and Methods

### 4.1. Study Site Description 

**Figure 6:**
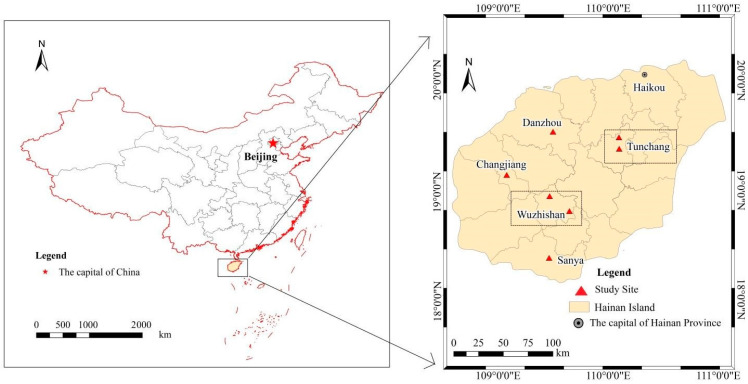
Experimental area and sampling sites.

The field study was conducted on Hainan Island (18°10′–20°10′ N, 108°37′–111°03′ E) in Hainan Province, southern China (Figure 6). Hainan Island is located in the tropical climate zone, and the climate is a typical tropical island monsoon climate. The annual average temperature on Hainan Island is 22.5–25.6 °C, and the annual average rainfall ranges from 923 to 2459 mm, with large spatial and temporal differences and an obvious seasonal distribution. The rainy season on Hainan Island ranges from May to October, which accounts for approximately 75–86% of the total precipitation in the whole year; the dry season is from November to April, and only 14–25% of the total rainfall occurs during this period. Most of the soil at the study sites is classified as yellow earth and red loam. The terrain of Hainan Island is high in the middle and low in the periphery, and it is composed of mountains, hills and platforms. 

### 4.2. Sample Collection and Analysis 

During the period from 29 August 2017 to 4 September 2017, the study area in Hainan Province was investigated. We designed 7 plots in Changjiang, Danzhou, Sanya, Tunchang and Wuzhishan based on different climate types, altitudes and habitats (Figure 6). There were two plots in the study sites of Tunchang and Wuzhishan, and only one plot in the remaining study area. Leaves from trees, herbs, vines and ferns were collected from 7 plots, which were located in primary or secondary forests far from areas of human activity. We selected all visible species within the plots, which included constructive species, such as *Gleditsia sinensis*, *Acacia confusa*, *Sapium sebiferum*, *Acronychia pedunculata*, *Sterculia lanceolata*, etc. Specifically, there was a distribution of 79, 86, 59, 84, and 106 species from Changjiang to Wuzhishan, respectively (Table 3). We collected 424 plant samples from 312 plant species in 109 families, including 14 unnamed tree species. For trees, mature leaves were collected from the middle and upper branches of 5 sampling trees in 4 different directions, and the leaves of 5 sample trees were mixed into one sample. For herbs, vines and ferns in the lower layer, mature leaves were collected from the top of five individuals. The samples were collected from open and sunny environments to avoid the influence of partial shading on plant growth. The samples were dried at 65 ℃ for 72 h and then ground and sifted through a 60-mesh sieve (0.25 mm diameter) for chemical analysis. The total C concentrations of leaf samples were determined by dry combustion using an elemental analyzer (Vario MAX CN Elemental Analyzer, Elementar, Germany). The leaf δ^13^C was determined using a British GV Instruments stable isotope mass spectrometer (GV IsoPrime-JB312, UK). The carbon isotopic ratios were reported in the delta notation relative to the Vienna–Pee Dee Belemnite (V-PDB) standard. δ^13^C is characterized by the following formula [16]:

(1) δCsample13=[(Rsample−Rstandard)/Rstandard]×1000

where δ^13^*C*_sample_ is the δ^13^C of the corresponding plant sample, ‰; *R*_sample_ and *R*_standard_ denote the ^13^C/^12^C molar ratios of the sample and the standard material (V-PDB), respectively.

### 4.3. Calculation of Intrinsic WUE 

The present study calculated the intrinsic WUE using the equation published by Farquhar et al. (1989) [14]. According to Farquhar et al. (1984) [16], the intrinsic WUE is calculated as follows:

(2) $$\mathrm{intrinsic}\;\mathrm{WUE}=A/g_{s}{=c}_{a}\left({1-\;c}_{i}/c_{a}\right)/1.6$$

The definitions of A, g_s_ and c_i_/c_a_ are shown in the introduction section. The carbon isotopic discrimination of C_3_ plants (Δ^13^C) is indicated by the following formula [14]:

(3) $$\Delta^{13}{C=(\delta }^{13}C_{\mathrm{air}}{\;-\;\delta }^{13}C_{\mathrm{plant}}{)/(1+\delta }^{13}C_{\mathrm{plant}}/1000)=a+\left(b\;-\;a\right)c_{i}/c_{a}$$

where δ^13^C_air_ and δ^13^C_plant_ are the δ^13^C of air and plants in ‰; a (= 4.4‰) is the diffusive discrimination of ^13^C in air through the stomata; and b (= 27‰) represents the net discrimination caused by carboxylation. Therefore, the intrinsic WUE can be calculated by leaf Δ^13^C [14,55]:

(4) $${\mathrm{intrinsic}\;\mathrm{WUE}=c}_{a}\left({b\;-\;\Delta }^{13}C_{\mathrm{plant}}\;\right)/1.6\left(b\;-\;a\right)\;$$

The data of c_a_ and δ^13^C_air_ in the formula were from Global Monitoring Laboratory (https://gml.noaa.gov, accessed on 20 December 2022).

### 4.4. Acquisition of Climate Data

The meteorological data including MAT, RST, MAP, RSP and WS on Hainan Island during 2016–2017 were collected from the China National Meteorological Data Center (Table A1). In addition, we used the published data of MAT and dew point data from the China National Meteorological Data Center to calculate RH by the Goff–Gratch equation. Additionally, based on the meteorological data from other meteorological stations on Hainan Island, the inverse distance weighted (IDW) method was used to fit the spatial variation map of climate data on Hainan Island for the study area, which still lacks climate data.

### 4.5. Statistical Analysis

For all statistical analyses, the LCC and intrinsic WUE data of each sample were grouped together according to plant functional types (i.e., trees, herbs, vines and ferns), location (i.e., Changjiang, Danzhou, Sanya, Tunchang and Wuzhishan), and altitudinal range (i.e., <200 m, 200–300 m, 300–400 m, 400–500 m, 400–500 m, 500–600 m and >600 m). The climate data of each study area during 2016–2017 were obtained by combining the inverse distance weight interpolation method of ArcGIS 10.6 and other research methods. IBM SPSS Statistics 25 was used for one-way ANOVA, multi-factor ANOVA, Pearson analysis, linear regression analysis and stepwise regression analysis.

One-way ANOVA was used to compare the differences in LCC and intrinsic WUE between each plant functional types. Multi-factor ANOVA was conducted to determine the effect of functional plant type, location, altitudinal range and their interaction on LCC and intrinsic WUE. Pearson analysis and linear regression were conducted to assess the relationships between the geographical and climatic factors with LCC and the intrinsic WUE. If there was more than one factor that had a significant impact on LCC and intrinsic WUE, multiple linear regression and stepwise regression analysis were used to analyze the combined effect of geographical and climatic factors on LCC and intrinsic WUE and to determine the main influencing factors.

## 5. Conclusions

In this study, LCC, intrinsic WUE and their relationships with geographical and climatic factors were investigated in 424 samples (from 312 plant species) from five sites on Hainan Island to explore the effect of global change on plant carbon content and intrinsic WUE. We found that the LCC of trees was significantly higher than that of herbs, vines and ferns. MAT was the main factor affecting the LCC of all plants and trees. Herbs, vines and ferns were less affected by geographical and climatic factors. Additionally, we found that there was no significant difference in the intrinsic WUE of different plant functional types. The plant intrinsic WUE in the study area was affected by multiple geoclimatic factors, and WS was the main driving factor. For trees, the intrinsic WUE was mainly affected by altitude; for ferns, the intrinsic WUE was mainly affected by altitude and RH; and for herbs and vines, geographical and climatic factors had little effect on the intrinsic WUE. This result indicates that in tropical regions with high temperature and high humidity, changes in geographical and climatic factors have an impact on plant intrinsic WUE, and for different plant functional types, the intrinsic WUE is affected by different geographical and climatic factors. Overall, the most important factor affecting leaf intrinsic WUE in the study area was WS. The present study played a crucial role in guiding the water management in tropical rainforest.

## Figures and Tables

**Table 1:** The results of multi-factor ANOVA.

	F	L	A	F × L	F × A	L × A
LCC	3.78 *	0.37	1.08	0.49	0.49	7.91 **
Intrinsic WUE	3.07 *	6.74 ***	4.42 **	1.73	0.91	2.56

Note: LCC, leaf carbon content; intrinsic WUE, intrinsic water use efficiency; F, plant functional types; L, location; A, altitudinal range. The data in the table are the F values, *, ** and *** indicate significant effects at the levels of *p* < 0.05, *p* < 0.01 and *p* < 0.001, respectively.

**Table 2:** The results of multiple linear regression analysis between intrinsic WUE and geographical and climatic factors.

	Methods	Factors Included in the Models	R^2^	*p*
All sample	Input	All factors	0.083	<0.001
	Stepwise	WS	0.024	0.001
Woody plants	Input	All factors	0.081	<0.001
	Stepwise	Altitude	0.024	0.006
Ferns	Input	All factors	0.810	0.048
	Stepwise	Altitude, RH	0.766	0.006

Note: LCC, leaf carbon content; intrinsic WUE, intrinsic water use efficiency. All factors included longitude, latitude, altitude, RH and WS. RH, relative humidity; WS, wind speed.

**Table 3:** Overview of the study area.

Study Site	Longitude (°E)	Latitude (°N)	Altitude (m)	MAT (℃)	MAP (mm)	Species
Changjiang	109°04′	19°07′	520–800	17.35	1563.12	79
Danzhou	109°29′	19°30′	137	23.86	1934.99	86
Sanya	109°27′	18°22′	387–568	23.47	1918.85	59
Tunchang	110°06′	19°21′	110–160	23.13	2105.15	84
	110°05′	19°27′				
Wuzhishan	109°38′	18°47′	490–520	22.80	2080.95	106
	109°28′	18°55′	260			

Note: MAT, mean annual temperature; MAP, mean annual precipitation.

## Data Availability

Not applicable.

## Appendix A

**Table A1:** Hainan meteorological station.

Study Site	Longitude (°E)	Latitude (°N)	Altitude (m)	MAT (℃)	MAP (mm)
Danzhou	109°29′	19°30′	170.20	23.86	1934.99
Sanya	109°27′	18°22′	6.00	25.33	1918.85
Haikou	110°15′	20°00′	64.70	24.08	1807.25
Dongfang	108°37′	19°06′	8.80	25.15	1255.55
Baisha	109°26′	19°14′	219.30	22.29	1928.77
Qiongzhong	109°50′	19°02′	253.00	21.98	2134.85
Qionghai	110°28′	19°14′	25.20	24.64	2066.99
Lingshui	110°02′	18°33′	13.90	23.87	2022.10

Note: MAT, mean annual temperature; MAP, mean annual precipitation.
